# Management and Rehabilitation of Dentigerous Cyst With 10-Year Follow-Up: A Case Report

**DOI:** 10.7759/cureus.67867

**Published:** 2024-08-26

**Authors:** Puneet Batra, Shweta G Batra, Dhruv Ahuja, Ravi Batra

**Affiliations:** 1 Orthodontics and Dentofacial Orthopaedics, Manav Rachna Dental College, Manav Rachna International Institute of Research and Studies, Faridabad, IND; 2 Private Practitioner, Dr Shweta’s Dental Clinic, New Delhi, IND; 3 Private Practitioner, Dentessence Dental Clinic, Gurgaon, IND

**Keywords:** rehabilitation, bone graft, enucleation, implant, dentigerous cyst

## Abstract

Odontogenic cysts are fluid-filled sacs arising from tooth-developing tissues within the jawbone, often causing swelling, pain, or displacement of teeth. A dentigerous cyst, specifically encapsulating the unerupted tooth's crown, can interfere with dental development by displacing teeth, causing resorption of adjacent teeth, and complicating dental or surgical interventions.

This case report represents a long-term follow-up of a 35-year-old male with a large mandibular dentigerous cyst extending to the lower right border of the mandible, associated with a horizontally impacted third molar in the lower right mandibular region. The treatment consisted of cone beam computed tomography(CBCT) evaluation, followed by enucleation of the cyst with the extraction of second and third molars, root canal treatment (RCT) of the first molar, followed by autogenous symphyseal bone graft placement. Eighteen months later, the implant was placed with respect to the missing second molar. After osseointegration, an implant-supported crown was placed.

## Introduction

Odontogenic cysts originate from the tissues involved in tooth development. These cysts can form within the jawbone or the soft tissues of the mouth and are characterized by fluid-filled sacs. They arise from the remnants of tooth-forming epithelium and can vary in size, location, and clinical behavior. Odontogenic cysts are significant in dental practice due to their potential to cause swelling, pain, displacement of teeth, and other complications if not properly managed. Understanding the different types and clinical implications of these cysts is essential for effective diagnosis and treatment [1].

Representing 14%-20% of all jaw cysts, dentigerous cysts are the second most common, having a male and mandibular pre-disposition. They are closely associated with tooth impactions, that is, the third molars and then maxillary canines, and sometimes found in association with odontomas or supernumerary teeth. While the exact pathogenesis is unknown, the proliferation of the reduced enamel epithelium due to osmotic pressure in a fluid-filled sac due to tooth impaction is thought to be one of the causes [2-5]. The origin of dentigerous cyst is often unclear; inflammation in the overlying tooth, when present, is suspected as a potential trigger for some dentigerous cysts [6,7]. They are typically identified during routine radiographic examination when a tooth fails to erupt within the normal time range and typically present as a unilocular radiolucency, with some of the larger cysts showing a scalloping multilocular pattern [8-10]. Dentigerous cysts, classified as benign odontogenic tumours by the World Health Organization (WHO), arise from odontogenic epithelium. Surgical treatment, ranging from enucleation for smaller cysts to surgical excision for complex cases, aims to remove the cyst completely. While marsupialization can be used for large cysts, laser intervention is still an evolving adjunct to traditional surgical methods [11].

Although they are usually asymptomatic, dentigerous cysts at times grow extensively, leading to complications and extensive treatment options including extraction of tooth buds or multiple teeth in addition to endangering vitality of surrounding teeth. Thus to prevent further damage, surgical elimination of the cyst still remains the best treatment option, which includes methods such as decompression, marsupialisation, and enucleation [2-5]. Nevertheless, the factors determining when to use or avoid these treatment modalities are not clearly established due to a lack of comprehensive literature with long-term follow-up.

This paper presents a long-term follow-up of a case involving a single implant placement for oral rehabilitation. The case includes a comprehensive clinical examination, cone beam computed tomography (CBCT) evaluation, and histopathological assessment, culminating in the enucleation of a large dentigerous cyst in the lower right mandibular region.

## Case presentation

A 35-year-old male sought evaluation at our private dental practice for a large radiolucent lesion and abnormal soft tissue growth, associated with an impacted lower right back tooth in the posterior mandible. Initially, the patient was referred to a plastic surgeon who proposed partial mandibular resection, which the patient opted not to undergo. The patient was generally healthy with an unremarkable medical history. The extraoral examination revealed swelling on the right side, causing facial asymmetry. Intraorally, the patient exhibited a substantial outward bulge on the buccal aspect of the lower right posterior region accompanied by restricted jaw movement.

**Figure 1:**
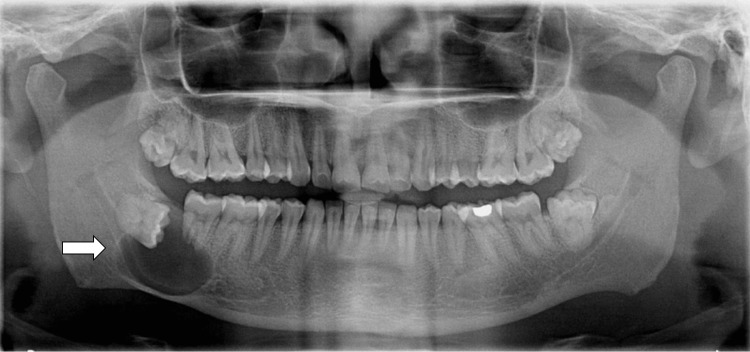
Pre-treatment OPG: showing unilocular radiolucency associated with the impacted tooth in the lower right mandibular region OPG: orthopantomogram

The orthopantomography revealed a unilocular cystic radiolucency that began at the resorbed root apex of the second molar, extended to the horizontally impacted third molar, and involved the right mandibular angle. The cyst was limited to the crown of the impacted third molar and extended down to the lower border of the mandible (Figure 1).

**Figure 2:**
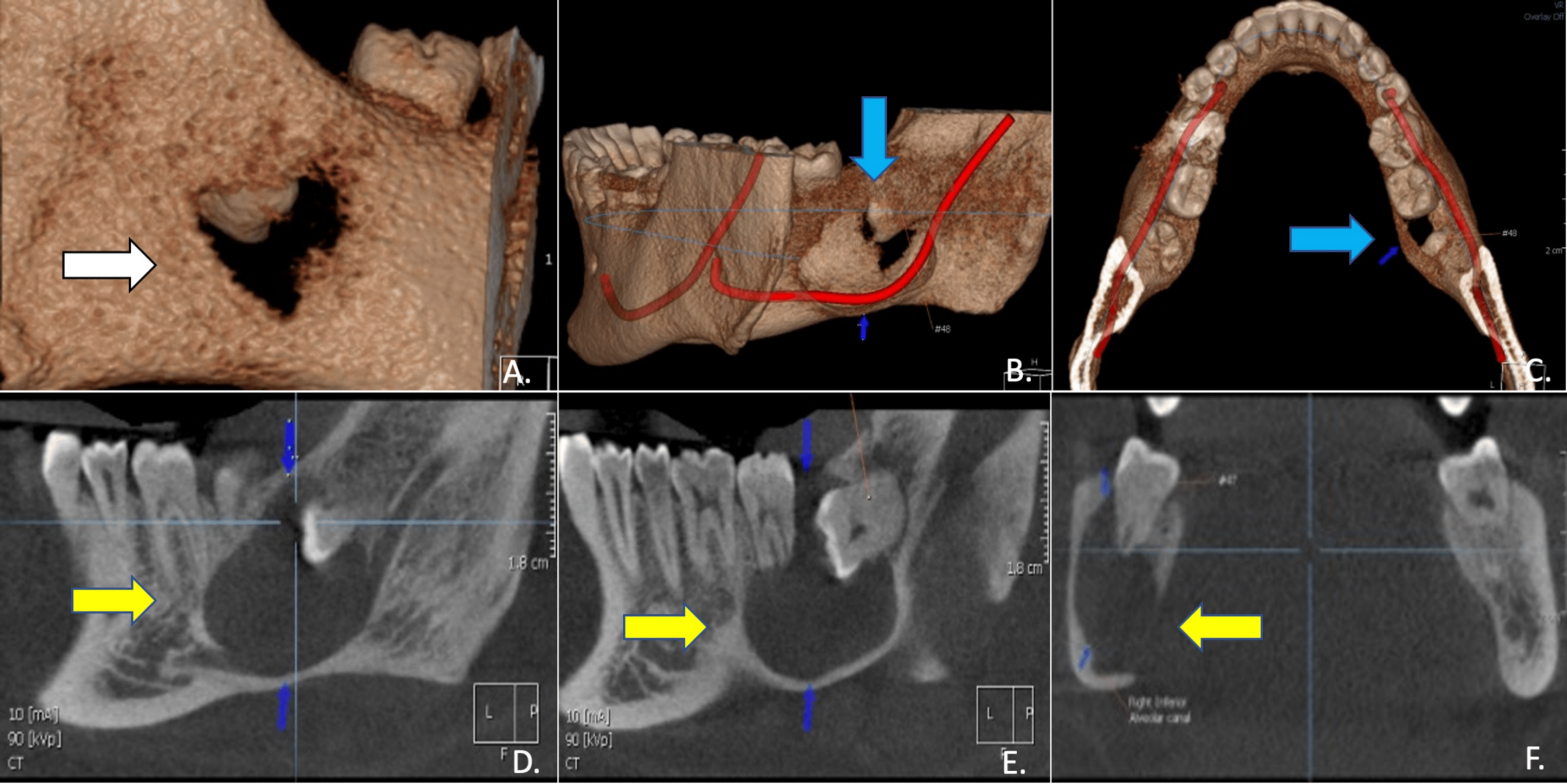
Pre-treatment CBCT: (A) cystic lesion and its association with mandibular bone (white arrow); (B,C) association of mandibular nerve and the cystic lesion (blue arrows); (D-F) radiolucent lesion associated with the impacted tooth, extending buccally and lingually to the mandibular bone margins (yellow arrows)

CBCT validated the panoramic radiograph results and further demonstrated a well-defined osteolytic lesion with expansive growth in the right posterior body and ascending ramus of the mandible, along with bucco-lingual enlargement in the right mandibular corpus. The lesion measured approximately 2.8cm x 1.4cm x 2.4cm respectively in greatest anteroposterior, transverse, and supero-inferior dimensions with distinct margins, corticated and attached to the cemento-enamel junction (CEJ) of the impacted third molar. The internal structure was homogenous and radiolucent with no obvious internal calcifications or septae. There was expansion and attenuation of the cortical bone on both the buccal and lingual surfaces, with intermittent breach of the lingual cortex and buccal cortex in the third molar region. There was inferior displacement of the right inferior alveolar canal with thinning and possibly partial effacement of the superior cortex and resultant scalloping of the mandibular inferior cortex was also seen. The lesion had caused apical root resorption of the right second molar as noted in the panoramic radiograph (Figure 2).

The preliminary diagnosis made after considering the radiological and clinical information was a dentigerous cyst; however, the remote possibility of a keratocystic odontogenic tumour was kept as a differential diagnosis. Blood investigation of the patient confirmed that serum alkaline phosphate and calcium levels were within their normal limits.

Treatment objectives

In the management of odontogenic cysts, several key objectives must be pursued to ensure comprehensive care and optimal outcomes. Histopathological investigation is essential for accurately diagnosing the type and nature of the cyst, which guides the subsequent treatment plan. Following diagnosis, the cyst is typically enucleated to remove the lesion and prevent further complications. After the cyst is removed, attention turns to rehabilitating any extracted teeth, which may involve dental implants or other restorative procedures to restore function and aesthetics. Finally, it is crucial to establish proper occlusion to ensure the patient's occlusion is functional and comfortable, thus promoting long-term oral health and preventing further dental issues. By addressing these objectives, a holistic approach to treating odontogenic cysts is achieved, leading to better patient outcomes.

Treatment progress

To ensure sufficient bony support and prevent potential fracture of the lower mandible due to the long-standing cyst, the decision was made to enucleate the cyst. The procedure was performed under general anaesthesia using an intraoral approach, and the cyst was completely removed. As the roots of the second molar were resorbed and the third molar was horizontally impacted, both teeth were extracted during the enucleation procedure. An autogenous symphyseal bone graft was placed for bone regeneration. Following the initial closure of the surgical site, the excised tissue was preserved in a 10% buffered formalin solution and sent for pathological evaluation. Root canal treatment was done in the first molar followed by a porcelain fused-to-metal (PFM) crown.

**Figure 3:**
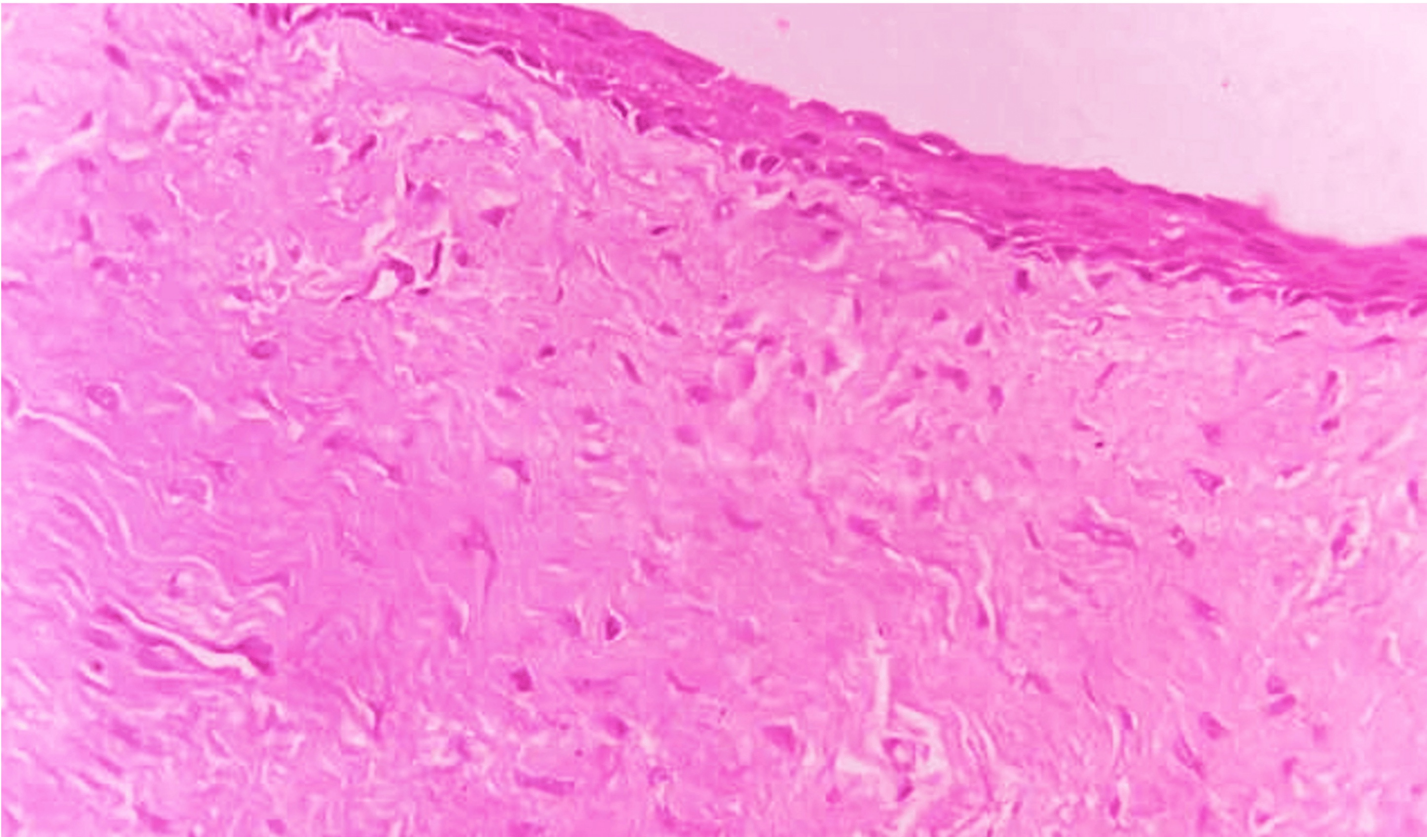
Histopathological examination of the cyst Magnification: 40x

Histopathological investigation revealed a cystic cavity lined by two- to three-layer thick epithelium with characteristics similar to reduced enamel epithelium. The epithelium connective tissue interface was flat. The underlying connective tissue capsule was fibrous with mild chronic inflammatory cells infiltrated. A few areas of extravasculated red blood cells (RBCs) were also appreciable along with peripheral areas exhibiting reactive bone formation. The histopathologic examination confirmed the diagnosis of a dentigerous cyst (Figure 3).

**Figure 4:**
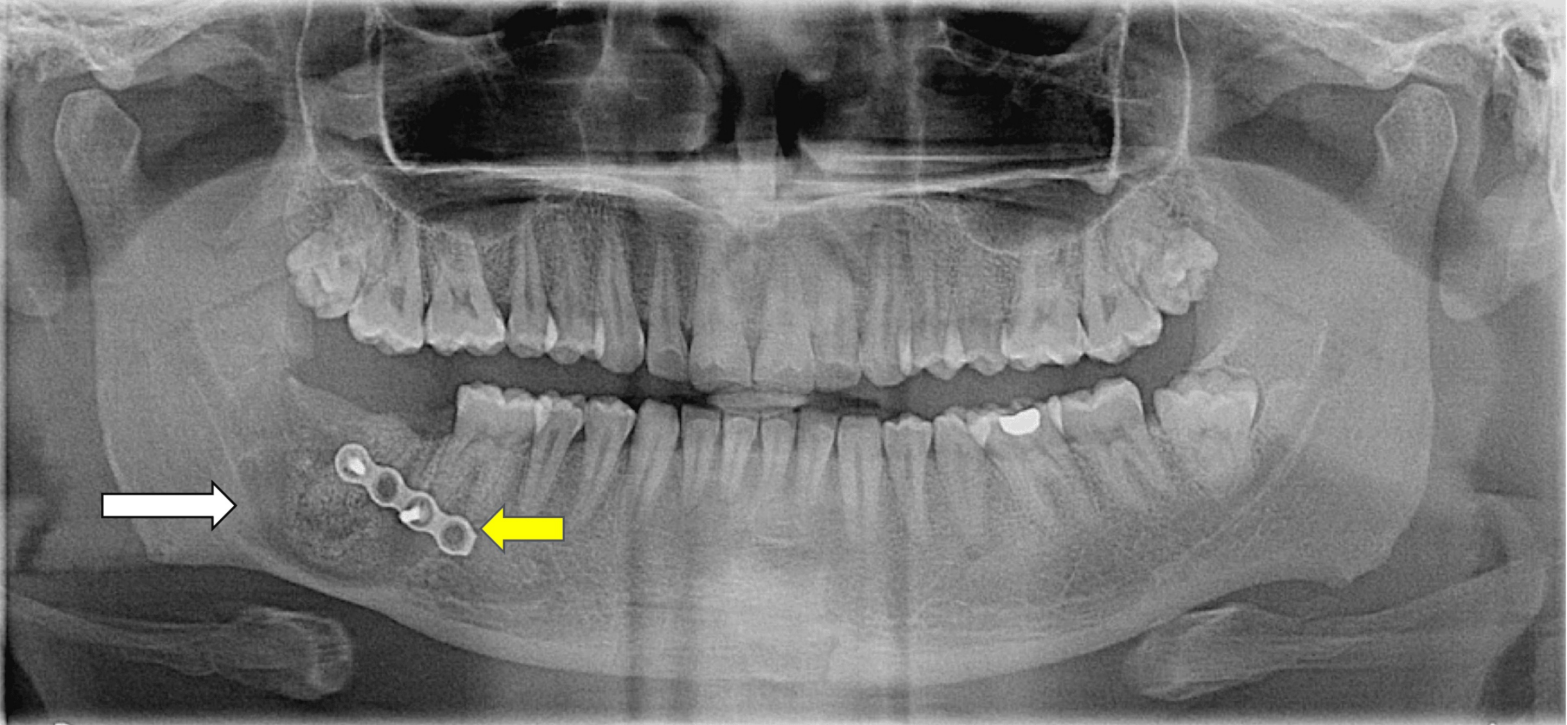
Enucleation of the cyst, with autogenous symphyseal bone graft placement (white arrow). A four-hole titanium bone plate was placed with two screws after enucleation for bone stability (yellow arrow)

No complications occurred intraoperatively. Antimicrobial and anti-inflammatory drugs were prescribed for the initial seven days following surgery. At the initial follow-up appointment, the patient reported experiencing paraesthesia on the lower right lip. After being prescribed neurogenic vitamins, the paraesthesia completely resolved by the fifth week post-surgery (Figure 4).

**Figure 5:**
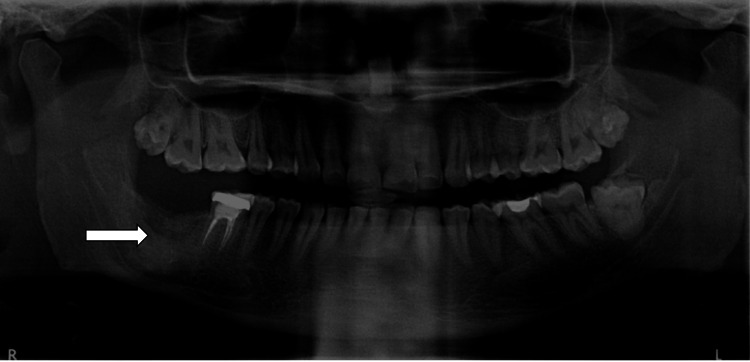
Radiographic evaluation at 18 months post-cyst enucleation and bone grafting. Bone regeneration was noticed in the mandibular right posterior region.

The radiographic follow-up conducted 18 months after enucleation and bone grafting displayed adequate bony regeneration at the surgical site (Figure 5).

**Figure 6:**
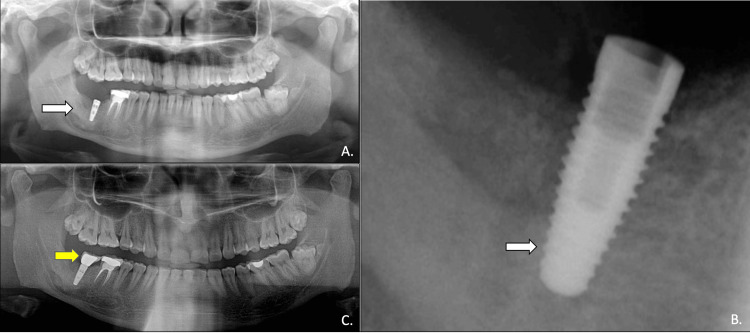
Radiographic assessment post-implant placement: (A,B) a titanium dental implant (Nobel Biocare 13mm) placed with surrounding bone regeneration (white arrows). (C) After six months of osseointegration, an implant-supported PFM crown was placed for the right second molar (yellow arrow) PFM: porcelain-fused-to-metal

There was a reduction in the size of the previously noted osteolytic/cystic lesion in the mandibular right posterior region, suggestive of endosteal new bone formation along the margins of the bone defect and complete re-cortication of the previously thinned-out adjacent inferior cortex of the mandible. These findings were suggestive of normal post-operative healing and remodelling. Following this, the bone plate was removed and one titanium dental implant (Nobel Biocare Replace RP 13mm; Nobel Biocare, Kloten, Switzerland) was placed in the right mandibular posterior region. Six months later, once the osseointegration occurred, a gingival former was placed for three weeks followed by an implant-supported ceramic crown (Figure 6).

Treatment follow-up

**Figure 7:**
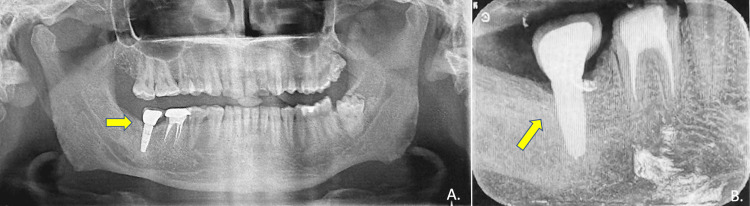
Post-treatment follow-up at 10 years: (A) orthopantomogram (OPG) shows adequate bone surrounding the implant and its relation to adjacent teeth; (B) intraoral periapical radiograph (IOPA) further confirmed implant success and bone remodelling

The implant was followed up for 10 years in the region previously affected by the dentigerous cyst. Over the decade, the implant exhibited stable integration with no signs of failure or cyst recurrence. Radiographic assessments showed satisfactory bone remodelling and graft success, while the patient reported improved oral function and no discomfort. This long-term success underscores the effectiveness of combining enucleation with grafting and implant placement in treating such cases (Figure 7).

## Discussion

In the present case, dentigerous cyst presented as a unilocular radiolucency in the right side of the mandible associated with horizontally impacted third molar and caused root resorptions of the adjacent right second molar, thereby leading to its extraction [12].

Cyst size plays a vital role in planning the treatment. Although small cysts are often easily enucleated and examined pathologically (excisional biopsy), this particular case involved a notably large cyst [13,14]. Managing larger lesions surgically usually involves decompression or marsupialization to ease the procedure [12,13]. However, it was decided to perform enucleation in view of the patient’s age, overall health status and possibility of pathological fracture if left untreated in the present case. In the present case, while the treated patient was an adult, but due to the cyst encroaching the mandibular canal, the enucleation of the cyst caused paraesthesia of the lower lip for a few weeks, which returned to normalcy later post surgery. 

In children, extensive cysts may cause damage to the tooth germs and, in addition, the teeth may also get devitalised during enucleation. Initially, decompression of the lesion is suggested to lessen its size, followed by enucleation surgery at a subsequent stage. Care must also be taken not to damage the adjacent vital structures including blood vessels and nerves. If an impacted tooth associated with the cyst is important aesthetically and augments occlusion, it should ideally be preserved during cyst removal, while a hopelessly decayed or impacted tooth such as the third molar often warrants extraction with cyst enucleation.

Autogenous symphyseal bone graft was placed after enucleation, helping in bone regeneration. Other allogeneic graft materials were noted in literature, which could be used for good implant bone contact. Tadjoedin et al. [15] and Cordioli et al. [16]. reported bioactive glass as a bone substitute in sinus lift procedures showed promising bone regeneration results, but these results were observed only when used alongside autologous bone. Carmagnola et al. [17] reported that the use of an osseoconductive bone xenograft resulted in new bone formation; however, histological evaluations demonstrated that there was no contact between the bone and the implant. In contrast, Valentini et al. found that both bone formation and bone-implant contact were achieved satisfactorily after using osseoconductive bone xenografts [18,19]. It is worth noting that the long-term success of dental implants is associated with their placement in natural, pre-existing bone [20-22]. So the best option of use of autogenous symphyseal graft was justified.

The retention or relapse of dentigerous cysts often stems from incomplete removal of the cyst lining, impacted tooth, or associated inflammatory tissue [6]; in this case, there was no evidence of recurrent cyst formation. The optimal timing for placing an implant after removing a large odontogenic cyst is usually determined by radiographic assessment [6,16]. In this case report, radiographs showed sufficient new bone formation 18 months after surgery to place an implant, aligning with findings reported by McCullagh et al. [23] typically, it takes six to eight months to make prosthesis after placing an implant in cases like these. In this case report, a crown was successfully placed on an implant in the lower right posterior region six months after the implant placement. However, there is limited research on successful implant placement after surgical removal of odontogenic cysts (Table 1). Nevertheless, to the best of our knowledge no long-term follow-up of dentigerous cyst with implant rehabilitation has been documented. Therefore, the present case report adds to the literature the use of autogenous symphyseal bone graft after enucleation of a large dentigerous cyst followed by implant placement.

**Table 1:** Literature review of dentigerous cyst cases

S. No.	Authors	Pathological lesion	Region of cyst	Treatment procedure	Time of implant placement	Graft used
1.	Bredfeldt et al. (1992) [10]	Odontogenic keratocyst	Upper right maxillary region-15, 16 and 17	Enucleation	36 months after enucleation	Iliac crest bone graft
2.	McCullagh et al. (1999) [23]	Odontogeneic keratocyst	Lower left mandibular region 36 and 37	Enucleation	18 months after enucleation	No graft
3.	Karamanis et al. (2006) [24]	Dentigerous cyst	Lower left mandibular region 37 and 38	Marsupialization	12 months after marsupialization	Alloplastic graft material placed
4.	Cakarer et al. (2011) [22]	Large dentigerous cyst	Lower left mandibular region 35, 36, 37 and 38	Decompression followed by enucleation	18 months after enucleation	No graft
5.	Aoki et al. (2018) [25]	Dentigerous cyst	Upper right maxillary region 13 and 12	Marsupialization	11 months after bone graft	Autogenous bone graft
6.	Correia et al.(2023) [26]	Odontogenic keratocyst	Upper right maxillary region 11,12,21 and 22	Enucleation	Six months after enucleation	Xenograft

## Conclusions

The case highlights the long-term follow-up of an adult patient with a large dentigerous cyst, which was addressed through enucleation. This case underscores the benefits of using enucleation followed by autogenous graft placement for bone regeneration. After the surgical procedure, an implant was successfully inserted. Combining implant insertion with graft placement after the enucleation of a cyst defect may offer a valuable alternative solution.
